# Trajectories of alcohol consumption in relation to all‐cause mortality in patients with cardiovascular disease: a 35‐year prospective cohort study

**DOI:** 10.1111/add.15850

**Published:** 2022-03-03

**Authors:** Chengyi Ding, Dara O'Neill, Annie Britton

**Affiliations:** ^1^ Research Department of Epidemiology and Public Health University College London London UK; ^2^ CLOSER, UCL Social Research Institute University College London London UK

**Keywords:** Alcohol, cardiovascular disease, former drinker, longitudinal, mortality, trajectory

## Abstract

**Background and Aims:**

Research into alcohol consumption and cardiovascular disease (CVD) patients' prognosis has largely ignored the longitudinal dynamics in drinking behaviour. This study measured the association between alcohol consumption trajectories and mortality risk in CVD patients.

**Design:**

Prospective cohort study.

**Setting:**

UK‐based Whitehall II Study.

**Participants:**

A total of 1306 participants with incident non‐fatal CVD (coronary heart disease/stroke) events.

**Measurements:**

Up to eight repeated measures of alcohol intake were available for each patient from the most recent assessment phase pre‐incident CVD and all subsequent phases post‐incident CVD, spanning up to three decades. Six trajectory groups of alcohol consumption were identified using group‐based trajectory modelling and related to the risk of all‐cause mortality, adjusting for demographics and changes in life‐style and health status.

**Findings:**

Three hundred and eighty deaths were recorded during a median follow‐up of 5 years after patients' last alcohol assessment. Compared with patients who consistently drank moderately (≤ 14 units/week), former drinkers had a greater risk of mortality (hazard ratio = 1.74, 95% confidence interval = 1.19–2.54) after adjustment for covariates. There was no significantly increased risk of mortality in long‐term abstainers, reduced moderate drinkers, stable or unstable heavy drinkers. Cross‐sectional analyses based only on drinking information at patients' last assessment found no significant differences in mortality risk for abstainers, former or heavy drinkers versus moderate drinkers.

**Conclusions:**

Cardiovascular disease patients who consistently drink ≤ 14 units/week appear to have a similar risk of mortality to those who are long‐term abstainers, which does not support a protective effect of moderate drinking on total mortality. Cardiovascular disease patients who stop drinking appear to have increased mortality risk compared with continuous moderate drinkers, but this may be linked to poor self‐rated health before cardiovascular disease onset.

## INTRODUCTION

Cardiovascular disease (CVD) is the leading cause of premature mortality and a major contributor to disability [1]. Globally, the number of prevalent CVD cases has increased rapidly since 1990, reaching 523 million in 2019 [2]. The association between moderate alcohol consumption and reduced risk of CVD is well‐documented and heatedly debated [3, 4, 5]. However, relatively few studies have focused on patients who have already experienced a CVD event and the effects that alcohol drinking may have on their subsequent health. A recent meta‐analysis suggests that drinking up to 105 g of ethanol per week is associated with lower risks of mortality and subsequent cardiovascular events than non‐drinking in those with established CVD [6]. It is noteworthy that this threshold is lower than the upper limits of drinking recommended in most current guidelines [7, 8, 9].

Similar to the critiques of studies on general populations [10, 11], the evidence among CVD patients is far from robust for several important reasons. First, most studies (11 of 14) included in the meta‐analysis only looked at the association cross‐sectionally, despite evidence that drinking behaviours change over time and that misclassification of alcohol intake has the potential to bias the risk estimates [12, 13]. Longitudinal prospective assessment of intake is needed to accurately measure long‐term exposure to alcohol, and this is particularly relevant when studying biological processes that cause chronic effects on health [14]. Secondly, in those few studies of CVD patients that did include longitudinal assessment of alcohol and subsequent health risks [15, 16, 17], the methodology used can be questioned. In most cases these studies categorized the patients into different drinking groups according to each patient's average intake during follow‐up, with no accounting for intra‐individual variation in drinking levels over time. Failure to capture such variation may result in over‐simplistic interpretation of alcohol use and consequent outcomes, as there is evidence from general population samples that unstable drinking patterns confer increased risks for coronary heart disease (CHD) and total mortality independent of average intake [18, 19, 20]. Thirdly, these studies often included former drinkers (who might have quit in response to ill health) in the non‐drinking group, which could erroneously lead to a suggested protective effect of drinking compared to non‐drinking. Indeed, when former drinkers were excluded from the meta‐analysis [6], the protective effect of moderate drinking on all‐cause mortality among CVD patients was eliminated. Fourthly, most studies also had a heterogeneous group of patients with incident or recurrent CVD events and did not adequately account for concurrent changes in other life‐style and health factors, such as smoking, which is associated both with levels of drinking and with mortality [21] and thus might confound the results.

It therefore remains unclear what advice should be given to CVD patients in terms of their alcohol consumption and subsequent prognosis. We contribute to this deficit in evidence using data with repeated measures of alcohol intake spanning up to three decades. We aimed to (1) describe the longitudinal trajectories of alcohol consumption in patients with incident CVD events, (2) link these trajectories to risk of all‐cause mortality and (3) compare these associations with cross‐sectional findings in the same cohort.

## METHODS

### Study design and population

The Whitehall II Study is an ongoing cohort study of 10 308 British civil servants aged 35–55 years at enrolment (phase 1), recruited from 20 London‐based offices during 1985–88 [22]. Phase 1 involved a clinical examination and a self‐administered questionnaire to collect information including demographics, health status and life‐style factors. Subsequent phases of data collection have alternated between questionnaire alone and questionnaire accompanied by a clinical examination. A linkage was made to the National Health Service (NHS) Hospital Episode Statistics database, which has been found valid for CVD ascertainment in the Whitehall II study [23, 24]. Incident CVD event was defined as a primary or secondary CHD/stroke diagnosis in the linked data set (using the procedure and International Classification of Diseases codes listed in Supporting information, Table S1), with additional cases identified on the basis of 12‐lead resting electrocardiogram recording (for CHD only) or self‐reports that had been verified with information from general practitioners or manual retrieval of medical records.

**FIGURE 1:**
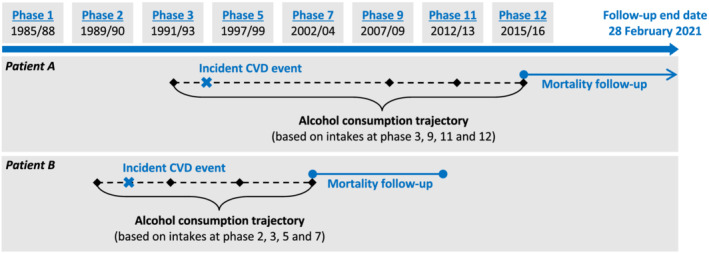
An illustration of study design. This figure provides two illustrative examples of how drinking trajectories were constructed for patient A, who had an incident cardiovascular disease (CVD) event in 1995 and was alive at the end of follow‐up, and for patient B, who had an incident event in 1990 and later died in 2012, using all available measures of alcohol intake for each patient starting from the most recent phase pre‐incident CVD. Duration of mortality follow‐up was calculated from date of each patient's last available alcohol assessment to the earliest of date of death, emigration or last follow‐up

Data used for the present analyses came from phases 1 (1985–88), 2 (1989–90), 3 (1991–93), 5 (1997–99), 7 (2002–04), 9 (2007–09), 11 (2012–13) and 12 (2015–16) of the Whitehall II study. We included participants who survived an incident CHD/stroke event during phases 1–12 and for whom repeated measures of alcohol were available (at least two measures, starting from the most recent phase pre‐incident CVD; Figure 1). Participants with previously diagnosed CHD/stroke or cancer at phase 1 were excluded from analyses to reduce reverse causality. The analysis was not pre‐registered and thus the results should be considered exploratory.

### Alcohol consumption

At each phase, participants were asked if they had consumed alcohol in the previous year, and if not whether they have always been non‐drinkers. Those who reported having consumed alcohol in the previous year were then asked about the number of alcoholic drinks they had consumed during the previous week. Drinks were converted into UK units of alcohol (1 unit equivalent to 8 g of ethanol) using a conservative estimate of 1 unit for each measure of spirits and small glass of wine, and 2 units for each pint of beer [25]. These converted measurements were summed to define the total weekly alcohol intake in units. We then categorized intakes at each phase into none, moderate (1–14 units/week) and heavy (> 14 units/week) to reflect the current UK drinking guidelines [26].

### Outcomes

All‐cause mortality was traced through the national mortality register. For each patient, follow‐up time began on the date of the patient's last available alcohol assessment and ended on the date of death, emigration, or 28 February 2021, whichever occurred first.

### Covariates

Socio‐demographic variables included age, sex and ethnicity. Socio‐economic position was defined using either current or last recorded employment grade as high, intermediate or low [27]. Health behaviours were assessed and comprised smoking (current, former or never), physical activity [meeting or below World Health Organization (WHO) recommendations] [28] and dietary behaviour (frequency of fruit and vegetables consumed in a week). Further medical information was obtained on self‐reported use of cardiovascular drugs, prevalent diabetes and hypertension. Covariates were assessed at the most recent phase pre‐incident CVD. To account for variability in the exposure assessment interval, the time difference between the date of first and last available alcohol assessment was calculated for each patient and included as a further covariate. Follow‐up observations on health behaviours and medical status were also derived from the same phase when the last available alcohol assessment was recorded.

### Statistical analysis

Group‐based trajectory modelling (GBTM), an extension of finite mixture modelling (FMM), was applied to identify groups of patients following different trajectories of alcohol consumption [29], with all available alcohol data (categorized into 0, 1–14 and > 14 units/week and coded as 0, 1 and 2, respectively) collected at the most recent phase pre‐incident CVD and from all subsequent phases post‐incident CVD (see Figure 1 for illustrative examples). Unlike growth mixture modelling (which is also FMM‐based), GBTM does not assume that the population is composed of discrete groups defined by different trajectories. Instead, GBTM uses groups as a statistical device for approximating the unknown distribution of trajectories in the population and is thus more appropriate for elucidating heterogeneity in alcohol use over time (as population differences in drinking trajectories are unlikely to be clear‐cut) [30]. We estimated trajectory models with three to six groups and for each group a polynomial function of time (up to second order) was considered, as suggested by previous research [31, 32]. The Bayesian information criterion was used to select optimal number and shape of groups. Patients were assigned to the group for which their posterior membership probability was highest (maximum‐probability rule). Model adequacy was evaluated using the recommended average posterior probability (AvePP ≥ 0.7 is indicative of a high assignment accuracy) [33].

Prior to undertaking inferential analyses, multiple imputation by chained equations was completed to address missing covariate data [34]. Outcome (the Nelson–Aalen hazard and outcome indicator) and exposure (alcohol intakes at each phase) variables were also included in the imputation model, but only observed values of these variables were used in the substantive analysis [35, 36]. We treated repeated measurements as distinct variables in the imputation model [37]. Simulation studies show that this approach performs well in similar longitudinal settings [38, 39]. Altogether, 100 imputations were run.

Hazard ratios (HRs) for all‐cause mortality in relation to drinking trajectories were estimated using Cox proportional hazards regression models. Models were first adjusted for age, sex and intake assessment interval (model 1), then additionally for ethnicity, socio‐economic position, health behaviours and medical status (model 2). Covariates in models 1 and 2 were from the most recent phase pre‐incident CVD. To account for changes in health behaviours as well as updates to medical status, further adjustment was made in model 3 for covariates (smoking, physical activity, dietary behaviour, use of cardiovascular drugs, prevalent diabetes and hypertension) assessed at the phase of last available alcohol assessment. Our reference group for analyses was stable moderate drinkers [40]. The proportional hazards assumption was tested using Schoenfeld residuals and found not to be violated (Supporting information, Figure S1).

We performed cross‐sectional analyses with drinking categories defined using only data from the last available alcohol assessment, so that findings from the main analyses (trajectory approach) can be compared to those that would have been obtained using the conventional approach in which exposure to alcohol was only assessed at one time‐point. Former drinkers were separated from abstainers in cross‐sectional analyses based on whether they reported at that phase to be always non‐drinkers.

Sensitivity analyses were conducted restricting analyses to either male patients, those with ≥ 3 alcohol measures, having CHD as first event or having complete‐case data. Previous research has suggested that the intake threshold associated with increased risk of mortality among CVD patients may be higher than 14 units/week [6, 41], so in exploratory *post‐hoc* analyses the average weekly intake during the assessment interval was calculated for each patient in the group of stable heavy drinkers. The group was then divided into two subgroups based on the group mean value of average weekly intakes, and their associations with mortality were examined. Additional *post‐hoc* analysis was conducted with further adjustment for concurrent changes in patients' self‐rated health (excellent/good, fair or poor). Self‐rated health has been shown to be a valid measure of overall health status as well as a predictor of mortality among participants of the Whitehall II study [42, 43]. Such analyses help to reveal whether changes in alcohol consumption occur as a consequence of worsening health. All analyses were performed using Stata version 15.1.

## RESULTS

### GBTM and sample characteristics

Of 10 308 Whitehall II participants, 178 were excluded due to a diagnosis of CHD/stroke or cancer before phase 1. A total of 1705 survived an incident CHD/stroke event from phases 1–12, 1306 of whom had repeated measures of alcohol and were included in this study.

**FIGURE 2:**
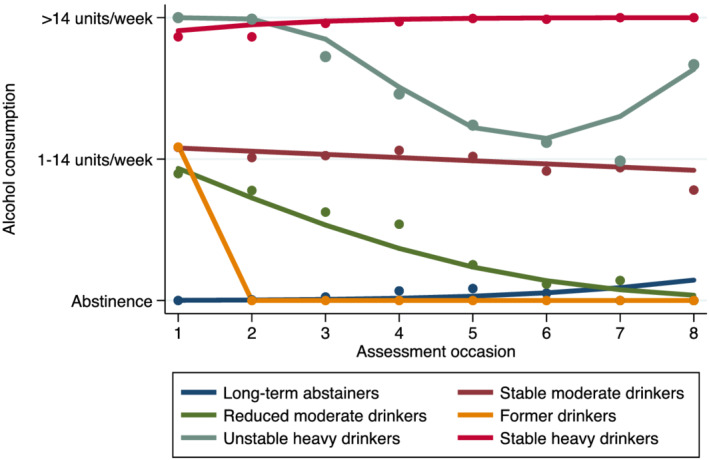
Alcohol consumption trajectories of the six groups identified using group‐based trajectory modelling. Assessment occasion 1 corresponds to the most recent phase pre‐incident CVD and assessment occasions 2–8 represent subsequent phases post‐incident cardiovascular disease (CVD). Solid lines indicate estimated trajectories and dot symbols indicate observed group means at each assessment occasion

In GBTM analysis, a six‐group model provided the best fit to the data (see Supporting information, Table S2 for model fit statistics) and showed adequate classification accuracy, with AvePP between 0.75–0.93. The identified trajectory groups are shown in Figure 2 (where occasion 1 corresponds to the most recent phase pre‐incident CVD), labelled *a posteriori* as: long‐term abstainers (15.5%), stable moderate drinkers (53.9%), reduced moderate drinkers (6.0%), former drinkers (6.3%), unstable heavy drinkers (8.5%) and stable heavy drinkers (9.8%). Overall, the resultant trajectories comprised a median assessment interval of 12.2 [interquartile range (IQR) = 7.0–18.0] years, with each patient contributing an average of four (IQR = 3–5) measures of alcohol.

Table 1 shows the characteristics of the study sample, as well as the proportion of missingness. Heavy drinkers (unstable or stable) were more likely to be male, of white ethnicity and high socio‐economic position; they were also more frequently past or current smokers at the most recent phase pre‐incident CVD. Across all trajectory groups, the proportions of patients currently smoking or meeting physical activity recommendations decreased from the most recent phase pre‐incident CVD to the phase of last available alcohol assessment. The prevalence of cardiovascular drug use, diabetes and hypertension increased during the same period.

**TABLE 1:** Patient characteristics by alcohol consumption trajectories

	Stable moderate drinkers	Long‐term abstainers	Reduced moderate drinkers	Former drinkers	Unstable heavy drinkers	Stable heavy drinkers	Overall
No. of patients	704 (53.9)	203 (15.5)	78 (6.0)	82 (6.3)	111 (8.5)	128 (9.8)	1306 (100)
Intake assessment interval, median (IQR) years	12.2 (6.9–17.9)	10.8 (5.2–17.2)	17.8 (12.9–23.3)	7.2 (4.2–12.6)	12.4 (6.4–18.2)	14.2 (11.2–19.1)	12.2 (7.0–18.0)
No. of alcohol measures, median (IQR)	4 (3–5)	3 (2–4)	5 (4–5)	3 (2–4)	4 (2–5)	4 (3–5)	4 (3–5)
At the most recent phase pre‐incident CVD							
Age, mean (SD) years	60.4 (8.9)	61.1 (9.2)	57.3 (8.1)	64.1 (9.1)	59.1 (9.1)	56.8 (7.9)	60.1 (9.0)
Male	574 (81.5)	97 (47.8)	44 (56.4)	53 (64.6)	107 (96.4)	123 (96.1)	998 (76.4)
Ethnicity							
White	635 (90.2)	131 (64.5)	65 (83.3)	69 (84.1)	109 (98.2)	123 (96.1)	1132 (86.7)
Non‐white	68 (9.7)	71 (35.0)	13 (16.7)	13 (15.9)	2 (1.8)	5 (3.9)	172 (13.2)
Missing	1 (0.1)	1 (0.5)	0 (0.0)	0 (0.0)	0 (0.0)	0 (0.0)	2 (0.2)
Socio‐economic position							
High	314 (44.6)	29 (14.3)	14 (17.9)	24 (29.3)	63 (56.8)	81 (63.3)	525 (40.2)
Intermediate	321 (45.6)	92 (45.3)	41 (52.6)	43 (52.4)	43 (38.7)	47 (36.7)	587 (44.9)
Low	69 (9.8)	82 (40.4)	23 (29.5)	15 (18.3)	5 (4.5)	0 (0.0)	194 (14.9)
Smoking status							
Never smoker	279 (39.6)	91 (44.8)	36 (46.2)	41 (50.0)	35 (31.5)	32 (25.0)	514 (39.4)
Ex‐smoker	302 (42.9)	61 (30.0)	21 (26.9)	33 (40.2)	56 (50.5)	67 (52.3)	540 (41.3)
Current smoker	79 (11.2)	30 (14.8)	14 (17.9)	7 (8.5)	17 (15.3)	21 (16.4)	168 (12.9)
Missing	44 (6.3)	21 (10.3)	7 (9.0)	1 (1.2)	3 (2.7)	8 (6.3)	84 (6.4)
Physical activity^a^							
Met recommendations	242 (34.4)	42 (20.7)	13 (16.7)	27 (32.9)	42 (37.8)	34 (26.6)	400 (30.6)
Below recommendations	422 (59.9)	142 (70.0)	55 (70.5)	53 (64.6)	66 (59.5)	84 (65.6)	822 (62.9)
Missing	40 (5.7)	19 (9.4)	10 (12.8)	2 (2.4)	3 (2.7)	10 (7.8)	84 (6.4)
Fruit/vegetable consumption							
≥ Daily	462 (65.6)	121 (59.6)	53 (67.9)	59 (72.0)	81 (73.0)	77 (60.2)	853 (65.3)
< Daily	204 (29.0)	65 (32.0)	18 (23.1)	23 (28.0)	25 (22.5)	42 (32.8)	377 (28.9)
Missing	38 (5.4)	17 (8.4)	7 (9.0)	0 (0.0)	5 (4.5)	9 (7.0)	76 (5.8)
Use of cardiovascular drugs							
Yes	248 (35.2)	84 (41.4)	23 (29.5)	30 (36.6)	40 (36.0)	39 (30.5)	464 (35.5)
No	437 (62.1)	110 (54.2)	54 (69.2)	52 (63.4)	69 (62.2)	85 (66.4)	807 (61.8)
Missing	19 (2.7)	9 (4.4)	1 (1.3)	0 (0.0)	2 (1.8)	4 (3.1)	35 (2.7)
Prevalent diabetes^b^							
Yes	73 (10.4)	50 (24.6)	10 (12.8)	13 (15.9)	16 (14.4)	17 (13.3)	179 (13.7)
No	612 (86.9)	145 (71.4)	67 (85.9)	69 (84.1)	93 (83.8)	107 (83.6)	1093 (83.7)
Missing	19 (2.7)	8 (3.9)	1 (1.3)	0 (0.0)	2 (1.8)	4 (3.1)	34 (2.6)
Prevalent hypertension^c^							
Yes	360 (51.1)	129 (63.5)	44 (56.4)	46 (56.1)	63 (56.8)	69 (53.9)	711 (54.4)
No	325 (46.2)	66 (32.5)	33 (42.3)	36 (43.9)	46 (41.4)	55 (43.0)	561 (43.0)
Missing	19 (2.7)	8 (3.9)	1 (1.3)	0 (0.0)	2 (1.8)	4 (3.1)	34 (2.6)
At the phase of last available alcohol assessment							
Smoking status							
Never smoker	259 (36.8)	83 (40.9)	29 (37.2)	31 (37.8)	31 (27.9)	27 (21.1)	460 (35.2)
Ex‐smoker	376 (53.4)	81 (39.9)	26 (33.3)	34 (41.5)	65 (58.6)	91 (71.1)	673 (51.5)
Current smoker	27 (3.8)	13 (6.4)	7 (9.0)	1 (1.2)	8 (7.2)	4 (3.1)	60 (4.6)
Missing	42 (6.0)	26 (12.8)	16 (20.5)	16 (19.5)	7 (6.3)	6 (4.7)	113 (8.7)
Physical activity^a^							
Met recommendations	142 (20.2)	22 (10.8)	11 (14.1)	12 (14.6)	23 (20.7)	27 (21.1)	237 (18.2)
Below recommendations	510 (72.4)	163 (80.3)	57 (73.1)	56 (68.3)	79 (71.2)	93 (72.7)	958 (73.4)
Missing	52 (7.4)	18 (8.9)	10 (12.8)	14 (17.1)	9 (8.1)	8 (6.3)	111 (8.5)
Fruit/vegetable consumption							
≥ Daily	500 (71.0)	125 (61.6)	43 (55.1)	57 (69.5)	78 (70.3)	87 (68.0)	890 (68.2)
< Daily	186 (26.4)	62 (30.5)	23 (29.5)	13 (15.9)	28 (25.2)	38 (29.7)	350 (26.8)
Missing	18 (2.6)	16 (7.9)	12 (15.4)	12 (14.6)	5 (4.5)	3 (2.3)	66 (5.1)
Use of cardiovascular drugs							
Yes	642 (91.2)	174 (85.7)	67 (85.9)	74 (90.2)	101 (91.0)	117 (91.4)	1175 (90.0)
No	60 (8.5)	28 (13.8)	11 (14.1)	8 (9.8)	10 (9.0)	11 (8.6)	128 (9.8)
Missing	2 (0.3)	1 (0.5)	0 (0.0)	0 (0.0)	0 (0.0)	0 (0.0)	3 (0.2)
Prevalent diabetes^b^							
Yes	239 (33.9)	109 (53.7)	47 (60.3)	46 (56.1)	40 (36.0)	52 (40.6)	533 (40.8)
No	465 (66.1)	94 (46.3)	31 (39.7)	36 (43.9)	71 (64.0)	76 (59.4)	773 (59.2)
Prevalent hypertension^c^							
Yes	609 (86.5)	173 (85.2)	66 (84.6)	77 (93.9)	97 (87.4)	111 (86.7)	1133 (86.8)
No	93 (13.2)	29 (14.3)	12 (15.4)	5 (6.1)	14 (12.6)	17 (13.3)	170 (13.0)
Missing	2 (0.3)	1 (0.5)	0 (0.0)	0 (0.0)	0 (0.0)	0 (0.0)	3 (0.2)

Values are numbers (percentages) unless stated otherwise.

^a^ Physical activity meeting WHO recommendations defined as ≥ 150 min of moderate‐intensity or ≥ 75 min of vigorous‐intensity activity per week.

^b^ Prevalent diabetes defined as reported doctor‐diagnosed diabetes, fasting blood glucose ≥ 7.0 mmol/l or use of antidiabetic drugs.

^c^ Prevalent hypertension defined as reported doctor‐diagnosed hypertension, systolic/diastolic blood pressure ≥ 140/90 mmHg or use of antihypertensive drugs. CVD = cardiovascular disease; IQR = interquartile range; SD = standard deviation.

### Alcohol consumption trajectories and all‐cause mortality

There were 380 deaths, with the median time from the last alcohol assessment to death being 5.0 (IQR = 4.4–5.7) years. Long‐term abstainers, stable and unstable heavy drinkers all had a similar risk of mortality as stable moderate drinkers after adjustment for all included covariates (Table 2). Compared to stable moderate drinkers, former drinkers had a higher risk of mortality after adjustment for covariates from the most recent phase pre‐incident CVD (model 2; HR = 1.84, 95% confidence interval (CI) = 1.26–2.68). The effect remained but was slightly attenuated in a maximally adjusted model with further adjustment for changes in other health behaviours and medical status (model 3; HR = 1.74, 95% CI = 1.19–2.54).

**TABLE 2:** Association between alcohol consumption and risk of all‐cause mortality

Alcohol consumption	No. of death	No. of patients	Hazard ratio (95% CI)		
			Model 1^a^	Model 2^b^	Model 3^c^
Trajectories					
Stable moderate drinkers	192	704	1.00 (Ref)	1.00 (Ref)	1.00 (Ref)
Long‐term abstainers	63	203	1.16 (0.86–1.56)	1.18 (0.87–1.62)	1.13 (0.83–1.55)
Reduced moderate drinkers	21	78	1.16 (0.73–1.84)	1.14 (0.72–1.83)	1.08 (0.67–1.73)
Former drinkers	35	82	1.77 (1.22–2.55)	1.84 (1.26–2.68)	1.74 (1.19–2.54)
Unstable heavy drinkers	34	111	1.28 (0.88–1.85)	1.24 (0.86–1.80)	1.25 (0.86–1.81)
Stable heavy drinkers	35	128	1.19 (0.83–1.72)	1.13 (0.78–1.64)	1.10 (0.76–1.60)
Categories based on single assessment only^d^					
Moderate drinkers	187	652	1.00 (Ref)	1.00 (Ref)	1.00 (Ref)
Abstainers	59	187	1.08 (0.80–1.46)	1.11 (0.81–1.52)	1.04 (0.76–1.44)
Former drinkers	78	245	1.23 (0.94–1.61)	1.24 (0.94–1.63)	1.16 (0.87–1.53)
Heavy drinkers	56	222	0.91 (0.67–1.23)	0.86 (0.63–1.17)	0.85 (0.62–1.15)

CI = confidence interval, Ref = reference; CVD = cardiovascular disease.

^a^ Adjusted for sex, age and intake assessment interval.

^b^ Additionally adjusted for ethnicity, socio‐economic position, smoking, physical activity, dietary behaviour, use of cardiovascular drugs, prevalent diabetes and hypertension, assessed at the most recent phase pre‐incident CVD.

^c^ Additionally adjusted for smoking, physical activity, dietary behaviour, use of cardiovascular drugs, prevalent diabetes and hypertension, assessed at the phase of last available alcohol assessment.

^d^ Drinking categories defined using intakes from the last available alcohol assessment.

### Cross‐sectional analyses

In cross‐sectional analyses, former drinkers had a point estimate of mortality risk greater than 1 when compared with moderate drinkers and adjusted for covariates from the most recent phase pre‐incident CVD (model 2; HR = 1.24, 95% CI = 0.94–1.63); this effect, however, was not statistically significant and was further attenuated in a maximally adjusted model (model 3; HR = 1.16, 95% CI = 0.87–1.53). There was little difference in mortality risk among abstainers and heavy drinkers compared to moderate drinkers (Table 2).

### Sensitivity analyses

Results of sensitivity analyses are in Supporting information, Table S3. The findings did not alter substantially when we restricted analyses to either male patients, those with ≥ 3 measures of alcohol or having CHD as first event. Similar associations were observed when using complete case data only.

### *Post‐hoc* analyses

Among the 128 stable heavy drinkers, mean weekly intake over the assessment interval was 30 [standard deviation (SD) = 12] units. Patients who died during follow‐up had higher weekly intakes than survivors (mean ± SD = 34 ± 14 units versus 28 ± 11 units, respectively). Compared to stable moderate drinkers, HR for all‐cause mortality was 1.53 (95% CI = 0.93–2.51) in stable heavy drinkers with weekly intakes > 30 units and 0.77 (95% CI = 0.45–1.30) in those with weekly intakes ≤ 30 units in maximally adjusted analysis (with adjustment for the same covariates listed in Table 2, model 3).

At the most recent phase pre‐incident CVD, long‐term abstainers had the lowest proportion of patients rating their health as excellent or good (55.7%), while unstable heavy drinkers had the highest (76.6%). The proportion decreased over the interval from the most recent phase pre‐incident CVD to last alcohol assessment in all trajectory groups (Supporting information, Table S4), with the greatest decrease seen in former drinkers (−36.8%, from 69.5 to 43.9%), followed by unstable heavy drinkers (−23.5%, from 76.6 to 58.6%,) and reduced moderate drinkers (−17.6%, from 65.4 to 53.8%). Further adjustment for changes in self‐rated health attenuated the associations between trajectories and all‐cause mortality (Supporting information, Table S5).

## DISCUSSION

In this inception cohort of patients with incident CVD events, we derived drinking trajectories with repeated assessments spanning up to 30 years and examined their association with subsequent risk of total mortality. Through iterative modelling that accounted for changing life‐style and health status, we found no evidence that patients who consistently consumed alcohol within the recommended limit of 14 units/week had a lower risk of mortality compared to long‐term abstainers. We also found that former drinkers had a greater mortality risk than stable moderate drinkers.

The elevated risk of mortality among former drinkers was only appreciable when considering long‐term drinking trajectories and was not significantly detected in our cross‐sectional analyses. Indeed, a large proportion of patients in this cohort did not have stable drinking trajectories following their incident CVD. Apart from those transiting from drinking to non‐drinking, this study also observed an overall decrease in alcohol intake over time among some continuers (reduced moderate drinkers and unstable heavy drinkers), as has also been reported elsewhere [16, 44]. The tendency towards desistance/lower levels of drinking with increasing age suggests that categorization of alcohol intake based on single time‐point measurements may be problematic, especially when applied to cohorts with long follow‐up periods and older participants. These highlight the importance of longitudinal measures and a life‐course approach in examining the effect of alcohol on health and our study should be replicated with other outcomes.

Our findings echo other research which suggests that former drinkers have poorer self‐perceived general health [45] and are at higher risk of experiencing adverse outcomes including CHD and overall mortality than moderate drinkers [18, 46]. As a reason for the higher risk seen in former drinkers, the sick‐quitter hypothesis proposes that a substantial number of former drinkers have quit drinking for health reasons [47, 48]. In line with this hypothesis, we found that former drinkers had a higher prevalence of poor self‐rated health than other groups at the most recent phase pre‐incident CVD and showed the biggest decrease in the proportion of patients reporting good to excellent health during follow‐up. The association for former drinkers was weakened following further adjustment for self‐rated health, suggesting that poorer general health may partially explain former drinkers' increased likelihood of death and perhaps may have driven the decision to abstain itself.

In the present study, no statistically significant protective effect was found in relation to consistent moderate drinking compared to long‐term abstinence. This concurs with general population studies measuring alcohol intake over time (collected either as repeated measures or as recall of past drinking levels) and mortality [49, 50, 51], as well as several Mendelian randomization studies where alcohol's cardioprotective effect has been tested and refuted [52, 53, 54]. Regarding CVD patients, longitudinal assessment of alcohol has been reported in two previous studies, where low levels of consumption were found to be associated with lower mortality [16, 17]. However, both studies have used a reference group composed of former drinkers and life‐time abstainers. The lower mortality risk for moderate drinking compared with non‐drinking could potentially be caused by a less healthy comparison group contaminated by sick quitters (as discussed above). Furthermore, the variety of reasons for which people abstain from drinking throughout life may introduce other biases. For instance, non‐drinkers in later life may include those who adopt life‐long teetotalism due to continual poor health [55]. In this study, only a small minority of CVD patients were long‐term abstainers. Notably, this group consisted mainly of women from a lower socio‐economic position with a higher prevalence of cardiometabolic risk factors and disease as well as poorer self‐rated health, a pattern that has also been reported in other study populations where alcohol use is normative [56, 57]. It has been suggested that members of this minority differ from drinkers on a number of health determinants and that unmeasured confounders may have contributed to the excess risk seen in this group [58, 59]. These motivated our choice of considering moderate drinkers as the reference group throughout this work and might explain the slightly increased point estimate for long‐term abstainers, despite the extensive level of adjustment in our analyses.

Although excessive drinking raises the risk of total mortality, the level from which this effect is evident is less clear. We assessed the impact of heavy drinking on CVD patients using the 14 units/week threshold advocated by the current UK guidelines and observed no elevated risk for those who consistently drank above this limit. Previous dose–response analyses using data from 83 general population cohorts have reported an intake threshold for increased mortality risk at ≥ 200 g/week (25 units/week) [41]. This agrees with the results of our *post‐hoc* analyses, where an increased risk was seen in stable heavy drinkers with higher average intakes (> 30 units/week). Clearly, the small number of patients within this group precludes any firm conclusion. Further data are therefore needed to explore alternative intake thresholds and validate the findings of the current study. In addition, heavy drinkers who remain in the cohort are likely to be ‘healthy survivors' or have safer drinking patterns and practices [10, 60]. At the most recent phase pre‐incident CVD, the proportion of patients drinking in excess of guidelines (36% male and 13% female) is lower than the recent estimates from Health Survey for England (39% male and 20% female aged 55–64 years) [61], which means that heavy drinkers may be under‐represented in our data set. These potential selections could have biased downwards the estimate of association between heavy intake and mortality risk, and thus caution is required when interpreting the lack of effect among heavy drinkers seen in our study.

There are other limitations that should be noted. First, our alcohol measures are self‐reported; however, self‐reports of drinking have shown reasonable levels of validity and reliability, especially when involving specified time‐frames (‘past week’ instead of ‘usual’ reference frames) and beverage‐specific questions [62, 63]. Comparison of alcohol consumption reported by the Whitehall II participants also suggests patterns similar to those in other UK cohorts [64]. Alcohol measures utilized in this study reflect intake only over the week immediately prior to each assessment, and may not be representative of participants' general consumption. Although this may introduce some exposure misclassification, the repeated assessment of alcohol over such a long period is unique. By integrating these repeated assessments, we were able to estimate trajectories, providing a more accurate account of longitudinal exposure than a cross‐sectional approach. Secondly, on the basis of maximum‐probability assignment rule, a level of uncertainty remains in individual‐level trajectory group membership. However, such uncertainty is unlikely to materially alter the profiles (characteristics and outcomes) that emerge from well‐fitting models such as the one in our GBTM analysis [33]. Because of power limitations restricting further refinement, we were unable to incorporate other drinking characteristics into the construction of trajectories. Additional data may provide insights into other drinking patterns, such as binge drinking, which could further clarify the observed mortality risk associated with unstable drinking trajectories. Relatedly, subgroup analyses (for example, in female or by age groups) were not possible due to the small number of patients in certain trajectory groups. In addition, participants in the Whitehall II study are not a representative sample of the general population; however, it has been shown that cardiometabolic‐related etiological evidence from this occupational cohort are broadly in agreement with those obtained from nationally representative cohorts [65]. Although we considered a wide range of covariates and accounted for their changes in the analyses, the possibility of residual confounding or confounding by unmeasured factors cannot be ruled out.

## CONCLUSION

In conclusion, this study has illustrated the dynamic and diverse nature of alcohol use in CVD patients and how long‐term drinking profiles are associated with their subsequent risk of death from all causes. By demonstrating the differing insights obtainable from cross‐sectional and repeated exposure assessment, this study has also confirmed the utility of taking a longitudinal approach in examining the association of alcohol with health outcomes. We found that CVD patients who consistently drank within the UK guidelines of 14 units/week had a similar risk of mortality as those who were continuous abstainers; therefore, this study does not support a protective effect of moderate drinking on total mortality. Patients who stopped drinking following incident CVD were at greater risk of mortality than continuous moderate drinkers; however, the former drinkers also had the highest proportion with poor self‐rated health before CVD onset and experienced the greatest degree of health deterioration during follow‐up. This study contributes to the dearth of evidence on health effects of alcohol consumption among CVD patients.

## DECLARATION OF INTERESTS

None.

## AUTHOR CONTRIBUTIONS

**Chengyi Ding:** Conceptualization; formal analysis. **Dara O'Neill:** Conceptualization; formal analysis; supervision. **Annie Britton:** Conceptualization; formal analysis; supervision.

## Supporting information




**Table S1** ICD and OPCS codes
**Table S2** Model fit statistics for estimation of alcohol consumption trajectories (group‐based trajectory modelling)
**Table S3** Sensitivity analyses for association between alcohol consumption trajectories and all‐cause mortality
**Table S4** Self‐rated health over the assessment interval by alcohol consumption trajectories
**Table S5** Association between alcohol consumption trajectories and all‐cause mortality with further adjustment for changes in self‐rated health
**Figure S1** Schoenfeld residuals
