# Supplementary material for: Trajectories of alcohol consumption in relation to all‐cause mortality in patients with cardiovascular disease: a 35‐year prospective cohort study
Source: Addiction. 2022 Mar 3;117(7):1920–30. doi: 10.1111/add.15850 (PMC9314067; doi:10.1111/add.15850)
Supplement: Supplementary file 1 — Table S1 ICD and OPCS codes Table S2 Model fit statistics for estimation of alcohol consumption trajectories (group‐based trajectory modelling) Table S3 Sensitivity analyses for association between alcohol consumption trajectories and all‐cause mortality Table S4 Self‐rated health over the assessment interval by alcohol consumption trajectories Table S5 Association between alcohol consumption trajectories and all‐cause mortality with further adjustment for changes in self‐rated health Figure S1 Schoenfeld residuals [file ADD-117-1920-s001.docx]

# Appendices

**Table S1** ICD and OPCS codes

| Coronary heart disease | |
| --- | --- |
| ICD-9 | 410-414 |
| ICD-10 | I20-I25 |
| OPCS4 | K40-K49, K50, K75, U19 |
| Stroke | |
| ICD-9 | 430, 431, 434, 436 |
| ICD-10 | I60, I61, I63, I64 |
| ICD=International Classification of Diseases, OPCS=OPCS Classification of Interventions and Procedures. | |

**Table S2** Model fit statistics for estimation of alcohol consumption trajectories (group-based trajectory modelling)

| Number of groups | Trajectory shapes ^a^ | BIC ^b^ | BIC ^c^ | AIC |
| --- | --- | --- | --- | --- |
| 3 | 1 1 1 | -5456.48 | -5450.46 | -5427.18 |
|  | 2 2 2 | -5476.88 | -5468.86 | -5437.81 |
| 4 | 1 1 1 1 | -5416.72 | -5408.69 | -5377.65 |
|  | 2 2 2 2 | -5430.96 | -5420.26 | -5378.86 |
| 5 | 1 1 1 1 1 | -5380.88 | -5370.85 | -5332.04 |
|  | 2 2 2 2 2 | -5402.02 | -5388.65 | -5336.90 |
| 6 | 1 1 1 1 1 1 | -5371.05 | -5359.02 | -5312.45 |
|  | 2 1 1 1 1 1 | -5357.42 | -5344.71 | -5295.55 |
|  | 1 2 1 1 1 1 | -5349.47 | -5336.77 | -5287.61 |
|  | 1 1 2 1 1 1 | -5369.88 | -5357.17 | -5308.01 |
|  | 1 1 1 2 1 1 | -5371.29 | -5358.58 | -5309.42 |
|  | 1 1 1 1 2 1 | -5371.29 | -5358.58 | -5309.42 |
|  | 1 1 1 1 1 2 | -5385.29 | -5372.59 | -5323.43 |
|  | 2 2 1 1 1 1 | -5353.73 | -5340.36 | -5288.61 |
|  | 2 1 2 1 1 1 | -5353.73 | -5340.36 | -5288.61 |
|  | 2 1 1 2 1 1 | -5360.41 | -5347.04 | -5295.30 |
|  | 2 1 1 1 2 1 | -5375.54 | -5362.17 | -5310.42 |
|  | 2 1 1 1 1 2 | -5379.57 | -5366.20 | -5314.45 |
|  | 1 2 2 1 1 1 | -5374.35 | -5360.98 | -5309.24 |
|  | 1 2 1 2 1 1 | -5335.95 | -5322.58 | -5270.83 |
|  | 1 2 1 1 2 1 | -5375.54 | -5362.17 | -5310.42 |
|  | 1 2 1 1 1 2 | -5353.73 | -5340.36 | -5288.61 |
|  | 1 1 2 2 1 1 | -5348.46 | -5335.09 | -5283.35 |
|  | 1 1 2 1 2 1 | -5354.81 | -5341.44 | -5289.69 |
|  | 1 1 2 1 1 2 | -5379.23 | -5365.86 | -5314.11 |
|  | ***1 1 1 2 2 1*** | ***-5332.79*** | ***-5319.42*** | ***-5267.67*** |
|  | 1 1 1 2 1 2 | -5345.52 | -5332.15 | -5280.40 |
|  | 1 1 1 1 2 2 | -5358.44 | -5345.07 | -5293.32 |
|  | 2 2 2 1 1 1 | -5376.03 | -5361.99 | -5307.65 |
|  | 2 2 1 2 1 1 | -5352.69 | -5338.65 | -5284.32 |
|  | 2 2 1 1 2 1 | -5352.72 | -5338.68 | -5284.35 |
|  | 2 2 1 1 1 2 | -5357.98 | -5343.94 | -5289.61 |
|  | 2 1 1 2 2 1 | -5340.41 | -5326.37 | -5272.04 |
|  | 2 1 1 2 1 2 | -5357.98 | -5343.94 | -5289.61 |
|  | 2 1 1 1 2 2 | -5357.98 | -5343.94 | -5289.61 |
|  | 2 1 2 2 1 1 | -5359.73 | -5345.69 | -5291.35 |
|  | 2 1 2 1 2 1 | -5352.69 | -5338.65 | -5284.32 |
| ^a^ Polynomial type for each group trajectory (0 intercept only, 1 linear, 2 quadratic).  ^b^ BIC for the total number of alcohol measurements (N=4973). A difference of 10 is strong evidence in favour of the model with a greater BIC; model with the highest (least negative) value of BIC has best fit.  ^c^ BIC for the total number of patients (N=1306).  BIC=Bayesian information criterion, AIC=Akaike information criterion. | | | | |

|  |  |  |  | ***(Continued)*** |
| --- | --- | --- | --- | --- |
| Number of groups | Trajectory shapes ^a^ | BIC ^b^ | BIC ^c^ | AIC |
| 6 | 2 1 2 1 1 2 | -5386.55 | -5372.51 | -5318.18 |
|  | 1 2 2 2 1 1 | -5379.47 | -5365.44 | -5311.10 |
|  | 1 2 2 1 2 1 | -5372.91 | -5358.87 | -5304.53 |
|  | 1 2 2 1 1 2 | -5340.51 | -5326.47 | -5272.13 |
|  | 1 2 1 2 2 1 | -5350.83 | -5336.79 | -5282.46 |
|  | 1 2 1 2 1 2 | -5340.51 | -5326.47 | -5272.13 |
|  | 1 2 1 1 2 2 | -5378.61 | -5364.57 | -5310.24 |
|  | 1 1 2 2 2 1 | -5329.48 | -5315.44 | -5261.11 |
|  | 1 1 2 2 1 2 | -5340.51 | -5326.47 | -5272.13 |
|  | 1 1 2 1 2 2 | -5357.90 | -5343.86 | -5289.53 |
|  | 1 1 1 2 2 2 | -5349.65 | -5335.61 | -5281.27 |
|  | 1 1 2 2 2 2 | -5376.33 | -5361.62 | -5304.70 |
|  | 1 2 1 2 2 2 | -5363.11 | -5348.40 | -5291.48 |
|  | 1 2 2 1 2 2 | -5382.80 | -5368.09 | -5311.17 |
|  | 1 2 2 2 1 2 | -5361.84 | -5347.13 | -5290.21 |
|  | 1 2 2 2 2 1 | -5388.56 | -5373.85 | -5316.93 |
|  | 2 1 1 2 2 2 | -5385.52 | -5370.81 | -5313.89 |
|  | 2 1 2 1 2 2 | -5390.78 | -5376.07 | -5319.15 |
|  | 2 1 2 2 1 2 | -5356.95 | -5342.24 | -5285.32 |
|  | 2 1 2 2 2 1 | -5390.76 | -5376.06 | -5319.14 |
|  | 2 2 1 1 2 2 | -5413.91 | -5399.20 | -5342.28 |
|  | 2 2 1 2 1 2 | -5356.95 | -5342.24 | -5285.32 |
|  | 2 2 1 2 2 1 | -5358.14 | -5343.43 | -5286.51 |
|  | 2 2 2 1 1 2 | -5380.28 | -5365.58 | -5308.65 |
|  | 2 2 2 1 2 1 | -5363.90 | -5349.19 | -5292.27 |
|  | 2 2 2 2 1 1 | -5356.91 | -5342.20 | -5285.28 |
|  | 1 2 2 2 2 2 | -5380.58 | -5365.21 | -5305.70 |
|  | 2 1 2 2 2 2 | -5367.36 | -5351.98 | -5292.48 |
|  | 2 2 1 2 2 2 | -5374.77 | -5359.40 | -5299.89 |
|  | 2 2 2 1 2 2 | -5373.73 | -5358.35 | -5298.84 |
|  | 2 2 2 2 1 2 | -5361.16 | -5345.79 | -5286.28 |
|  | 2 2 2 2 2 1 | -5362.34 | -5346.97 | -5287.46 |
|  | 2 2 2 2 2 2 | -5377.97 | -5361.93 | -5299.83 |
| ^a^ Polynomial type for each group trajectory (0 intercept only, 1 linear, 2 quadratic).  ^b^ BIC for the total number of alcohol measurements (N=4973). A difference of 10 is strong evidence in favour of the model with a greater BIC; model with the highest (least negative) value of BIC has best fit.  ^c^ BIC for the total number of patients (N=1306).  BIC=Bayesian information criterion, AIC=Akaike information criterion. | | | | |

**Table S3** Sensitivity analyses for association between alcohol consumption trajectories and all-cause mortality

| Alcohol consumption trajectories | No. of death | No. of patients | Hazard ratio (95% CI) ^a^ |
| --- | --- | --- | --- |
| ***Restricting to patients with ≥ 3 alcohol measures (n=990)*** | |  |  |
| Stable moderate drinkers | 130 | 533 | 1.00 (Ref) |
| Long-term abstainers | 31 | 136 | 1.02 (0.66-1.57) |
| Reduced moderate drinkers | 20 | 77 | 1.10 (0.67-1.81) |
| Former drinkers | 17 | 45 | 1.78 (1.04-3.05) |
| Unstable heavy drinkers | 23 | 80 | 1.33 (0.84-2.09) |
| Stable heavy drinkers | 33 | 119 | 1.23 (0.83-1.83) |
| ***Restricting to patients with CHD (n=1212)*** | |  |  |
| Stable moderate drinkers | 175 | 645 | 1.00 (Ref) |
| Long-term abstainers | 60 | 189 | 1.22 (0.88-1.68) |
| Reduced moderate drinkers | 20 | 75 | 1.11 (0.68-1.81) |
| Former drinkers | 28 | 73 | 1.53 (1.01-2.33) |
| Unstable heavy drinkers | 34 | 106 | 1.35 (0.93-1.97) |
| Stable heavy drinkers | 35 | 124 | 1.12 (0.77-1.63) |
| ***Restricting to male patients (n=998)*** |  |  |  |
| Stable moderate drinkers | 162 | 574 | 1.00 (Ref) |
| Long-term abstainers | 29 | 97 | 0.97 (0.64-1.46) |
| Reduced moderate drinkers | 11 | 44 | 0.90 (0.48-1.70) |
| Former drinkers | 23 | 53 | 1.56 (0.98-2.48) |
| Unstable heavy drinkers | 33 | 107 | 1.22 (0.83-1.78) |
| Stable heavy drinkers | 34 | 123 | 1.06 (0.72-1.55) |
| ***Complete case data only (n=1061)*** |  |  |  |
| Stable moderate drinkers | 170 | 579 | 1.00 (Ref) |
| Long-term abstainers | 48 | 161 | 0.97 (0.69-1.37) |
| Reduced moderate drinkers | 14 | 49 | 1.10 (0.63-1.92) |
| Former drinkers | 26 | 64 | 1.54 (1.00-2.37) |
| Unstable heavy drinkers | 29 | 93 | 1.08 (0.72-1.61) |
| Stable heavy drinkers | 29 | 104 | 0.99 (0.66-1.48) |
| ^a^ Adjusted for the same covariates listed in Table 2 Model 3.  CI=confidence interval, Ref=reference, CHD=coronary heart disease. | | | |

**Table S4** Self-rated health over the assessment interval by alcohol consumption trajectories

|  | Stable moderate drinkers | Long-term abstainers | Reduced moderate drinkers | Former drinkers | Unstable heavy drinkers | Stable heavy drinkers |
| --- | --- | --- | --- | --- | --- | --- |
| ***Self-rated health at the most recent phase pre-incident CVD, n (%)*** | | | | |  |  |
| Excellent/good | 516 (73.3) | 113 (55.7) | 51 (65.4) | 57 (69.5) | 85 (76.6) | 92 (71.9) |
| Fair | 128 (18.2) | 61 (30.0) | 16 (20.5) | 18 (22.0) | 21 (18.9) | 24 (18.8) |
| Poor | 23 (3.3) | 12 (5.9) | 5 (6.4) | 7 (8.5) | 2 (1.8) | 2 (1.6) |
| Missing | 37 (5.3) | 17 (8.4) | 6 (7.7) | 0 (0.0) | 3 (2.7) | 10 (7.8) |
| ***Self-rated health at last available alcohol assessment, n (%)*** | | | |  |  |  |
| Excellent/good | 481 (68.3) | 96 (47.3) | 42 (53.8) | 36 (43.9) | 65 (58.6) | 90 (70.3) |
| Fair | 167 (23.7) | 60 (29.6) | 19 (24.4) | 28 (34.1) | 33 (29.7) | 29 (22.7) |
| Poor | 38 (5.4) | 28 (13.8) | 9 (11.5) | 8 (9.8) | 9 (8.1) | 8 (6.3) |
| Missing | 18 (2.6) | 19 (9.4) | 8 (10.3) | 10 (12.2) | 4 (3.6) | 1 (0.8) |
| CVD=cardiovascular disease. | | | | | | |

**Table S5** Association between alcohol consumption trajectories and all-cause mortality with further adjustment for changes in self-rated health

| Alcohol consumption trajectories | No. of death | No. of patients | Hazard ratio (95% CI) ^a^ |
| --- | --- | --- | --- |
| Stable moderate drinkers | 192 | 704 | 1.00 (Ref) |
| Long-term abstainers | 63 | 203 | 1.03 (0.75-1.41) |
| Reduced moderate drinkers | 21 | 78 | 1.04 (0.64-1.67) |
| Former drinkers | 35 | 82 | 1.53 (1.04-2.25) |
| Unstable heavy drinkers | 34 | 111 | 1.13 (0.78-1.64) |
| Stable heavy drinkers | 35 | 128 | 1.12 (0.78-1.63) |
| ^a^ Adjusted for the same covariates listed in Table 2 Model 3 ***PLUS*** self-rated health assessed at the most recent phase pre-incident CVD and at the phase of last available alcohol assessment.  CI=confidence interval, Ref=reference, CHD=coronary heart disease. | | | |

**Figure S1** Schoenfeld residuals

*Notes: test of proportional hazards (PH) assumption is based on a test of non-zero slope in a generalised linear regression of the scaled Schoenfeld residuals on a function of time; a non-zero slope is an indication of a violation of PH assumption.*

a. All-cause mortality for models using alcohol consumption trajectories

b. All-cause mortality for models using alcohol consumption categories based on single assessment only
